# Identifying and responding to family adversity in Australian community and primary health settings: a multi-site cross sectional study

**DOI:** 10.3389/fpubh.2023.1147721

**Published:** 2023-09-13

**Authors:** Teresa Hall, Leanne Constable, Sarah Loveday, Suzy Honisett, Natalie Schreurs, Sharon Goldfeld, Hayley Loftus, Renee Jones, Andrea Reupert, Marie B. H. Yap, Sue Woolfenden, Alicia Montgomery, Kim Dalziel, Cate Bailey, Glenn Pringle, Jane Fisher, Suzie Forell, Valsamma Eapen, Ric Haslam, Lena Sanci, John Eastwood, Harriet Hiscock

**Affiliations:** ^1^Centre for Community Child Health, Murdoch Children's Research Institute, Parkville, VIC, Australia; ^2^Department of Paediatrics, The University of Melbourne, Parkville, VIC, Australia; ^3^Melbourne School of Population and Global Health, The University of Melbourne, Parkville, VIC, Australia; ^4^School of Educational Psychology and Counselling, Faculty of Education, Monash University, Clayton, VIC, Australia; ^5^Turner Institute for Brain and Mental Health, School of Psychological Sciences, Monash University, Clayton, VIC, Australia; ^6^Sydney Institute Women, Children and their Families, Sydney Local Health District, Croydon, NSW, Australia; ^7^Discipline of Paediatrics, University of New South Wales, Sydney, NSW, Australia; ^8^Sydney Medical School, University of Sydney, Sydney, NSW, Australia; ^9^Sydney Institute Women, Children and Their Families, Sydney Local Health District, Camperdown, NSW, Australia; ^10^Health Economics Unit, Melbourne School of Population and Global Health, The University of Melbourne, Parkville, VIC, Australia; ^11^Innovation and Community Care, IPC Health, Wyndham Vale, VIC, Australia; ^12^Global and Women's Health, Monash University, Clayton, VIC, Australia; ^13^Health Justice Australia, Darlinghurst, Sydney, NSW, Australia; ^14^School of Law, University of New South Wales, Sydney, NSW, Australia; ^15^Infant Child and Adolescent Psychiatry, University of New South Wales, Sydney, NSW, Australia; ^16^Department of General Practice, The University of Melbourne, Parkville, VIC, Australia; ^17^Health Services and Economics, Centre for Community Child Health, Murdoch Children's Research Institute, Parkville, VIC, Australia; ^18^Health Services Research Unit, The Royal Children's Hospital, Parkville, VIC, Australia

**Keywords:** community health service, integrated care, integrated health service, primary care, child mental health, childhood adversity, integrated hub

## Abstract

**Background:**

Unaddressed family adversity has potentially modifiable, negative biopsychosocial impacts across the life course. Little is known about how Australian health and social practitioners identify and respond to family adversity in community and primary health settings.

**Objective:**

To describe, in two Australian community health services: (1) the number of adversities experienced by caregivers, (2) practitioner identification of caregivers experiencing adversity, (3) practitioner response to caregivers experiencing adversity, and (4) caregiver uptake of referrals.

**Methods:**

Survey of caregivers of children aged 0–8 years attending community health services in Victoria and New South Wales (NSW). Analysis described frequencies of caregiver self-reported: (1) experiences of adversity, (2) practitioner identification of adversity, (3) practitioner response to adversity, and (4) referral uptake. Analyses were sub-grouped by three adversity domains and site.

**Results:**

349 caregivers (Victoria: *n* = 234; NSW: *n* = 115) completed the survey of whom 88% reported experiencing one or more family adversities. The median number of adversities was 4 (2–6). Only 43% of participants were directly asked about or discussed an adversity with a practitioner in the previous 6 months (Victoria: 30%; NSW: 68%). Among caregivers experiencing adversity, 30% received direct support (Victoria: 23%; NSW: 43%), and 14% received a referral (Victoria: 10%; NSW: 22%) for at least one adversity. Overall, 74% of caregivers accepted referrals when extended.

**Conclusion:**

The needs of Australian families experiencing high rates of adversity are not systematically identified nor responded to in community health services. This leaves significant scope for reform and enhancement of service responses to families experiencing adversity.

## 1. Introduction

### 1.1. Family adversity as a priority mental health issue

Family adversity is an umbrella term for negative experiences and conditions that include childhood maltreatment, parental mental illness, family violence, socio-economic deprivation, bullying and discrimination (1). Family adversity has detrimental impacts on the health and wellbeing of families and their children across the life course, increasing the lifetime risks of anxiety, depression, suicidality, obesity, cancer, and heart disease (2–4). Mitigating these impacts early in life is a public mental health priority because these conditions are common, preventable, and inequitably distributed. More than half of all Australian children have been exposed to two or more adversities by the age of 11 years, with adversities clustering in children from low socioeconomic backgrounds and minority ethnic and linguistic groups (5).

### 1.2. Identifying and responding to family adversity in health care

The systematic identification of, and response to, family adversity through universal and primary health services represents a critical opportunity to promote robust and equitable child and family mental health and wellbeing (6). In the United States, recent movements to implement standardized screening for Adverse Childhood Experiences (ACEs) have generated an emerging body of evidence on the acceptability and feasibility of identifying and responding to family adversity in health care settings. There are mixed findings from a family perspective. Some studies have found that families report that being asked about their experiences of adversities and/or their broader social needs (i.e., food insecurity) is acceptable and desirable (7–9). Conversely, caregivers in other studies found discussing adversities uncomfortable, emotionally difficult and/or re-traumatizing (10).

Intervention studies that have encouraged practitioners to ask about and/or screen for adversity have highlighted major challenges in changing practice. These include a lack of health practitioner confidence and competence to identify adversity and the perception that identifying or responding to adversity is outside their scope of practice (7, 11). Health practitioners also tend not to ask about adversity when they lack information about local referral pathways or when these services are not readily available (11).

Little is known about current practices in identifying and responding to family adversity in health services in Australia, where the model of health services and system infrastructure differs significantly from the US model investigated in most studies to date. The Australian health care system is underpinned by universal health insurance (Medicare) and includes a complex mix of federal, state and territory, and non-government services. Community health services offer free or low-cost care and are often the first point-of-contact for families with the health system (12). While community health services operate differently across Australian jurisdictions, in general, these services often include primary care and emphasize localization to best meet the needs of priority populations. As such, understanding the characteristics and needs of caregivers attending these services could provide a useful “snapshot” of adversity experienced by low-income Australian families and the corresponding opportunities for intervention.

### 1.3. The current study

This study investigated current practice in identifying and responding to adversity in families attending two Australian community health services. Both health services are part of a larger project that aims to co-design, test, evaluate, and scale integrated Child and Family Hubs, with an upstream focus on identifying and responding to family adversity (13). The services are in low socioeconomic, metropolitan areas that serve families who may experience various adversities, namely, Wyndham Vale in Victoria and Marrickville in New South Wales (NSW). Each service was chosen based on pre-existing relationships with the research team and is broadly representative of community health services in terms of a focus on delivery of low or no cost care in a social model of health.

#### 1.3.1. Wyndham Vale

The City of Wyndham is a metropolitan local government area (LGA) in the outer South-Western suburbs of Greater Melbourne and is home to 74 276 families with children (14). Over sixty percent of Wyndham's children aged 0–4 years have two parents born overseas (14). The Australian Early Development Census (AEDC) estimates that twenty-one percent of children in Wyndham are developmentally vulnerable in one or more developmental domains compared to Victoria state average of 19.9% (15).

IPC Health General Practitioner (GP) Super Clinic Wyndham Vale was selected as the Victorian site. At the time of data collection, Wyndham Vale was a community and primary health service that hosted a range of co-located but not integrated practitioners, including GPs, practice nurses, pediatricians, maternal child health services (general and enhanced), allied health and financial counseling (for gambling only). The Wyndham Vale service is independently registered and managed by IPC Health.

#### 1.3.2. Marrickville

Marrickville is a suburb in the Inner-West of Sydney, located within the Sydney Local Health District (SLHD). The Marrickville-Sydenham-Petersham Statistical Area Level 3 (SA3) in the Inner West Council local government area has a population of 54 824 individuals and 13 284 families (16). Marrickville is a culturally diverse suburb consisting of both low and high density residential, commercial and light industrial areas. Despite gentrification over time, Marrickville remains an area with significant diversity in housing prices with great dispersion around the median—in addition to high rates of private rental and social housing.While the proportion of children developmentally vulnerable on one or more domains of AEDC in Marrickville was lower than the NSW state average (16.9% and 21.2%, respectively), local geospatial mapping within the SLHD identified sub-areas of significant disadvantage within Marrickville using Socio-Economic Index for Areas (SEIFA) data (17).

Marrickville Community Health Center, nested within the established community and allied health service at Marrickville, was selected as the second site for this study. The district also has an existing partnership with the Healthy Homes and Neighborhoods (HHAN) services for vulnerable families (18), but Marrickville Community Health Center has not previously been a site for targeted place-based or co-located intervention via this initiative. HHAN involves training and capacity building opportunities in family partnerships models of practice and care coordination. The Marrickville service hosts child and family nurses, allied health practitioners, pediatricians, social workers, midwives, psychologists, mental health services, psychiatrists, and oral health services. The Marrickville service is registered and managed by SLHD.

### 1.4. Study aims

To describe and compare caregivers across the two community health services in terms of the:

1. number of adversities experienced,
2. extent to which practitioners asked about or discussed these adversities (identification),
3. extent to which practitioners spent extra time working through these adversities with the caregiver (response: direct) or made a referral to address these adversities (response: referral), and
4. uptake of these referrals.

## 2. Methods

### 2.1. Sample and procedures

This study analyzed baseline survey data collected as part of a mixed methods process and outcomes evaluation of two integrated Child and Family Hubs (13). Caregivers were recruited from November 2021 to June 2022 by researchers in the waiting rooms of each service or via a mail out to the services' client databases. Eligible participants were pregnant women or caregivers of children aged 0–8 years who understood written and spoken English and accessed any of the universal and/or specialist services provided in each service i.e., GPs, pediatricians, child and family health nurses, etc. Participants provided written informed consent before completing the online survey, or verbal consent for phone-administered surveys. See Supplementary material A for the baseline survey. Participants received a $25 honorarium for completing the survey which took ~30 min. A total of 380 caregivers consented (Victoria: *n* = 265, NSW: *n* = 115). Several participants were excluded because they did not submit the survey (Vic: *n* = 31; NSW: *n* = 1) and did not have a child aged 0–8 years (NSW only: *n* = 1), leaving a sample of 349 participants (Victoria: *n* = 234, NSW: *n* = 115). Ethical approval was received from the Royal Children's Hospital Human Research Ethics Committee (HREC/ 62866/RCHM-2020) and Sydney Local Health District (HREC/ 62866/RCHM-2020, 2020/STE05572) for the Victoria and NSW sites, respectively. This study is a prospectively registered trial: ISRCTN55495932.

### 2.2. Measures

Table 1 presents the variable definition and operationalization in the study.

**Table 1:** Key variable definition and operationalization.

**Variable**	**Survey question (s)**	**Survey responses included in analysis**
Adversities outside the home domain	Over the past 6 months, how many of the following four challenges have you felt concerned about affecting your child/ren or family: • Not enough contact with or support from others for yourself • Not enough money for everyday things such as food, clothing or bills • Problems with housing like worrying about keeping your home, having to share your home, or having a house that's too crowded, or in need of repair • Someone in the family having problems finding or keeping a job, insecure employment or a job that is not family friendly	0, 1, 2, 3, 4
Adversities inside the home domain	Over the past 6 months, how many of the following eight challenges have you felt concerned about affecting your child/ren or family: • My own physical health or disability or that of another family member inside or outside of my home • My own challenging feelings like feeling emotional, depressed, angry, anxious, exhausted or even having strange thoughts such as harming myself or others • The way that I (or my partner) manage my child/ren's daily routines, physical needs and their behavior • My child/ren being left to look after themselves too much, not having their needs met, or being given too much responsibility for their age • Someone in my family drinking alcohol or using drugs • Conflict or tension between members of my family • My child/ren might be seeing or exposed to behavior within the family/at home that frightens them like threats, bullying, yelling, screaming, putting people down, hitting, slapping, kicking, or punching • Someone in the family having problems finding or keeping a job, insecure employment or a job that is not family friendly	0, 1, 2, 3, 4, 5, 6, 7, 8
Societal adversities	Over the past 6 months, how many of the following three challenges have you felt concerned about affecting your child/ren or family: • Issues with visa or immigration for someone in my family or myself. • My own or a family member's court appearances as a defendant, being on bail, parole or spending time in prison. • Someone in my family has experienced discrimination or harassment such as bullying, racism	0, 1, 2, 3, 4
Identification	In the past 6 months, has any staff member at IPC Health asked you about any of these challenges?	•Yes • No, but I brought it up myself
Response: direct support	Did the staff member spend extra time with you talking about or working through the challenges?	•Yes
Response: referral	Did the staff member connect you to a different service or organization for support with these challenges	• Yes
Uptake of referral	Did you go to the different service or organization?	• Yes, I've been • Yes, I've been to some but not others • No, I'm still on a waiting list

#### 2.2.1. Experiences of adversity

Caregivers rated the frequency with which they had experienced adversities in each of three domains. *Adversities outside the home* included challenges with social support, finances, housing and employment. *Adversities inside the home* included challenges with family physical health or disability, mental health, parenting, relationships, family violence, alcohol and drugs, child neglect and child abuse. Adversities outside and inside the home domains were derived from the Parent Engagement Resource (19). In accord with Karatekin and Hill's (1) expanded definition of adversity, we added *societal adversities* as a third domain to capture challenges with visas or migration, interaction with the criminal justice system, and discrimination or harassment (see Table 1).

#### 2.2.2. Identifying and responding to adversity

We asked caregivers questions about identification and response within each adversity domain. *Identification* was defined as a caregiver report of practitioners “directly asking about adversity or discussing adversities” raised by the caregiver. Identification was defined in this way to capture a relational approach to care in which practitioners directly ask about adversity as well as actively listen to issues raised by caregivers (see Table 1).

Only those caregivers who indicated that the adversity had been identified were asked to complete the response questions. *Response variables* were defined as practitioners: (i) spending extra time working through the adversity i.e., *direct support* and (ii) referring the caregiver to a different service i.e., *referral* (see Table 1). Caregivers in receipt of a referral reported their *uptake of the referral* i.e., defined as having attended or being on the waitlist at the referred service.

#### 2.2.3. Caregiver characteristics

Caregivers reported caregiver age, gender, Aboriginal and/or Torres Strait Islander status, country of birth, main language spoken at home, education level, postcode, number of children in the household, and study child's age and gender. We used postcode to determine family SEIFA based on census data (20) calculated as quintiles.

### 2.3. Data analysis

Formal statistical testing was not conducted because this was an exploratory study to describe and compare identification of, and response to, adversity across the two community health services. *Total adversity* was defined as the total frequency of adversities across all three domains (range 0 to 15), to reflect literature suggesting no differences in child outcomes between adversity types (2). An *adversity across domains* variable was created to count the presence of adversities in 0, 1, 2, or 3 domains. Participant data were excluded from both these variables if there was one or more scores missing in any of the adversity domains (Vic: *n* = 21, 9.0%, NSW: *n* = 5, 4.3%). *Any adversity* variables were created for each identification and response question. Participant data were included if there were one or more scores present in any of the three domains for each variable.

The denominator for the identification variable was the total sample. To measure unmet need, the denominator for response variables was the number of the total sample who had experienced one or more adversities in the corresponding domain. The denominator for referral uptake variable was all participants who received a referral.

### 2.4. Participant characteristics

Table 2 displays the sociodemographic characteristics of the 349 caregivers included in analysis. Overall, most Victorian and NSW caregivers were female (*n* = 295, 85%) with an average age of 36.4 years (*SD* = 6.7) and lived with two or more adults in their household (*n* = 310, 89%). Compared to NSW caregivers, more Victorian caregivers spoke a language other than English at home (Vic: *n* = 96, 41%; NSW: *n* = 12, 11%), were born outside of Australia (Vic: *n* = 145, 62%; NSW: *n* = 33, 29%), did not attain university educational qualifications (Vic: *n* = 114, 49%; NSW: *n* = 25, 22%), had more children in their households(2 or more) (Vic: *n* = 164, 70%; NSW: *n* = 52, 46%), and more children with disabilities (Vic: *n* = 80, 35%; NSW: *n* = 16, 14%).

**Table 2:** Descriptive statistics on caregiver and child sociodemographic characteristics.

**Characteristics**	**Victoria**	**NSW**	*p* value^*^
*N^#^*	234	115	
**Caregiver**			
Age in years, mean (SD)	35.8 (6.5)	37.6 (7.1)	0.023^∧^
**Gender** ***n*** **(%)**			
Woman	189 (81)	106 (92)	0.004
Man	42 (18)	8 (7)	
Non-binary/gender diverse	0 (0)	1 (1)	
Prefer not to say	2 (1)	0 (0)	
**Aboriginal and/or Torres Strait Islander** ***n*** **(%)**			
Yes	8 (3)	4 (3)	1.000
No	225 (97)	111 (97)	
**Country of birth** ***n*** **(%)**			
Australia	88 (38)	82 (71)	< 0.001
Other	145 (62)	33 (29)	
**Main language spoken at home** ***n*** **(%)**			
English	136 (59)	102 (89)	< 0.001
Other	96 (41)	12 (11)	
**Highest level of education** ***n*** **(%)**			
Year 11 or below	34 (15)	4 (3)	< 0.001
Year 12 or equivalent	34 (15)	10 (9)	
Diploma or equivalent	46 (20)	10 (9)	
Bachelor degree or above	119 (51)	90 (78)	
Don't know	0 (0)	1 (1)	
**SES by postcode, ranking by state**^a^ ***n*** **(%)**			
Lowest 20%	158 (73)	61 (53)	< 0.001
Second lowest 20%	0 (0)	0 (0)	
Middle 20%	0 (0)	18 (16)	
Second highest 20%	19 (9)	12 (10)	
Highest 20%	39 (18)	24 (21)	
**Number of adults living in household** ***n*** **(%)**			
1	30 (13)	9 (8)	0.206
2 or more	204 (87)	106 (92)	
**Number of children in the household** ***n*** **(%)**			
0 (Caregiver currently pregnant)	2 (1)	3 (3)	< 0.001
1	68 (29)	59 (52)	
2	101 (43)	43 (38)	
3 or more	63 (27)	9 (8)	
**Child in the household with a disability** ***n*** **(%)**			
Yes	80 (35)	16 (14)	< 0.001
No	151 (65)	98 (86)	
Child age^2^ in years, median (IQR)	4.3 (1.8, 6.6)	0.6 (0.1, 3.7)	< 0.001^∧^
**Child gender**^2^ ***n*** **(%)**			
Female	81 (35)	57 (50)	0.017
Male	147 (64)	57 (50)	
Prefer not to answer	1 (0.4)	1 (0.9)	
**Number of adversities inside the home** ***n*** **(%)**			
0	47 (21)	21 (19)	0.028
1	33 (15)	32 (28)	
2	53 (23)	15 (13)	
3	40 (18)	21 (19)	
4	27 (12)	15 (13)	
≥5	27 (12)	9 (8)	
**Number of adversities outside the home**^c^ ***n*** **(%)**			
0	57 (26)	47 (42)	0.037
1	71 (32)	28 (25)	
2	48 (22)	21 (19)	
3	17 (8)	9 (8)	
4	27 (12)	7 (6)	
**Number of societal challenges**^c^ ***n*** **(%)**			
0	164 (75)	100 (91)	0.004
1	44 (20)	9 (8)	
2	5 (2)	1 (1)	
3	6 (3)	0 (0)	
**Total number of adversities reported** ***n*** **(%)**			
0	24 (10)	14 (12)	0.469
1	20 (9)	14 (12)	
2	22 (9)	16 (14)	
3	30 (13)	18 (16)	
4	31 (13)	15 (13)	
≥5	86 (37)	33 (29)	
**Number of domains of adversity experienced** ***n*** **(%)**			
0	24 (11)	14 (13)	0.002
1	42 (20)	37 (34)	
2	105 (49)	52 (47)	
3	42 (20)	7 (6)	

^#^Fluctuations in cell count for some variables are due to missing data.

^*^Fisher's exact test; ^∧^Welsh's *t*-test (variance of means).

^a^Quintiles of SEIFA Index of Relative Socio-economic Advantage and Disadvantage, by state (Australia Bureau of Statistics (ABS). 2016).

^b^caregivers with more than one child responded in relation to one child in their family they were most concerned about.

^c^adversities outside the home were challenges with social support, finances, housing and employment; adversities inside the home were challenges with physical health or disability, mental health, parenting, child neglect, alcohol and drugs, relationships, family violence and child abuse; societal adversities were challenges with visa or migration, the criminal justice system and discrimination or harassment.

Across both sites, caregivers reported high frequencies of *total adversity* (Vic: *n* = 189, 89%; NSW: *n* = 96, 87%, see Table 2). Eighty percent of caregivers reported experiencing at least one form of adversity inside the home (Vic: *n* = 180, 79%, NSW: *n* = 92, 81%). Over half reported experience of adversity outside the home (Vic: *n* = 163, 74%, NSW: *n* = 65, 58%). However, most caregivers did not report experiencing societal adversities (Vic: *n* = 55, 25%, NSW: *n* = 10, 9%). Almost 40% of the Victorian sample had experienced five or more adversities of any type compared to almost 30% of the NSW sample (*n* = 89 and *n* = 33, respectively). More than half of each sample had experienced two or more *adversities across domains*.

### 2.5. Identification of and response to adversity

Table 3 summarizes the proportions of identification of and response to adversity for caregivers at the Victorian and NSW sites. Higher proportions of caregivers from NSW reported that they were asked about or discussed an adversity with a practitioner than Victorian caregivers (Vic: *n* = 70, 30%; NSW: *n* = 78, 68%). Victorian and NSW caregivers reported greater identification of *adversities occurring inside and/or outside the home* than *societal adversities*.

**Table 3:** Identification and response to adversity across adversity types.

	**Victoria**		**NSW**		
	***N** ^∧^*	***n*** **(%)**	***N** ^∧^*	***n*** **(%)**	***p*** **-value** ^*^
**Identification** ^a^					
Any adversity	233	70 (30)	115	78 (68)	< 0.001
Adversity inside the home^d^	227	51 (22)	114	65 (57)	< 0.001
Adversity outside the home^d^	228	42 (18)	115	56 (49)	< 0.001
Societal adversities^d^	220	12 (5)	105	7 (7)	0.624
**Response: direct support** ^b^					
Any adversity	210	49(23)	101	43 (43)	0.001
Adversity inside the home^d^	180	34 (19)	92	37 (40)	< 0.001
Adversity outside the home^d^	163	24 (15)	65	15 (23)	< 0.001
Societal adversities^d^	55	8 (15)	10	0 (0)	0.017
**Response: referral** ^c^					
Any adversity	210	21 (10)	101	22 (22)	0.008
Adversity inside the home^d^	180	15 (8)	92	17 (18)	< 0.001
Adversity outside the home^d^	163	12 (7)	65	8 (12)	< 0.001
Societal adversities^d^	55	1 (2)	10	0 (0)	0.680
**Uptake of referral**					
Any adversity	19	15 (79)	19	13 (68)	0.714
Adversity inside the home^d^	14	12 (86)	17	10 (59)	0.132
Adversity outside the home^d^	12	8 (67)	7	5 (71)	1.000
Societal adversities^d^	1	1 (100)	0	0 (0)	-

^∧^Denominator for response variables was the number of the total sample who had responded as experiencing one or more adversities, fluctuations in cell count for some variables are due to missing data.

^*^Fisher's exact test.

^a^Identification: asked about or discussed adversity.

^b^Response: direct support i.e., spent time working through adversity.

^c^Response: referral to a different service for support.

^d^adversities outside the home were challenges with social support, finances, housing and employment; adversities inside the home were challenges with physical health or disability, mental health, parenting, child neglect, alcohol and drugs, relationships, family violence and child abuse; societal adversities were challenges with visa or migration, the criminal justice system and discrimination or harassment.

Less than half of caregivers with adversities at both sites reported receiving direct support for any adversity experienced (Vic: *n* = 49, 23%, NSW: *n* = 43, 43%). Greater direct support was provided to caregivers experiencing an adversity in NSW than in Victoria for *adversities inside the home* (Vic: *n* = 34, 19%; NSW: *n* = 37, 40%) and *outside the home* (*n* = 15, 23% and *n* = 24, 15%, respectively). NSW caregivers experiencing adversities reported greater receipt of direct support for *adversities inside the home* than for *societal adversities*. Across both sites, low proportions of caregivers experiencing adversities reported receiving a referral to a different service or organization. Victorian caregivers reported fewer referrals than in NSW (Vic: *n* = 21, 10%, NSW: *n* = 22, 22%). Most of the Victorian and NSW caregivers reported taking up referrals received for any type of adversity (*n* = 15, 79% and *n* = 13, 68%, respectively).

## 3. Discussion

Our study described patterns in the identification of, and response to, family adversity in two Australian community health services. In both sites, caregivers reported experiencing high levels of adversities inside (e.g., challenges with parental mental illness, parenting, alcohol and drugs, family violence) and outside the home (e.g., challenges with social support, finances, housing, employment). Both Victorian and NSW caregivers reported substantially higher frequencies of two or more adversities (78% and 75%, respectively) than the national average of 52.8% based on the Longitudinal Study of Australian Children (LSAC) (5). This was expected because our study purposively sampled families from community health centers which typically service low-income families. The Victorian caregivers in this study also reported more risk factors for disadvantage detected than those in the LSAC data i.e., they had lower average educational attainment and were more likely to be members of minority ethnic and linguistic groups (5). Overall, the caregivers sampled through our study are broadly representative of the Wyndham and Marrickville—Sydenham—Petersham SA3 populations based on Australian Census 2021 data (14, 16). Specifically, the mean age, country of birth, main language spoken at home, and child gender are comparable, with the exception that fewer participants (3%) identified as Aboriginal and Torres Strait Islander in the Victorian site than the general Wyndham population (9%); however, this trend was the opposite for the Marrickville site (3% compared to 1.6% in the general Marrickville population) (14, 16). In sum, our study captured a group of families experiencing multiple adversities, whose service experiences have important insights for early intervention and prevention service planning in community health.

The trends in the identification of, and response to, adversity highlight a large unmet need for support for families experiencing adversity accessing these community health services. In Victoria, only one-third of caregivers were identified by practitioners as experiencing any type of adversity. Identification of any adversity in NSW by practitioners was higher (70%). However, there were low reported rates of practitioner response—either through direct support or referral—in both sites. Only one-third of NSW caregivers experiencing one or more types of adversity were provided with direct support and only 20% in Victoria. Referral uptake was relatively high, suggesting that the referrals made were deemed appropriate by the recipient caregivers. Taken together, these findings highlight an opportunity to better engage families about adversities in the two community services to optimize child and family mental health and wellbeing through addressing the broader social determinants of mental health (6).

The relatively enhanced identification of adversity in NSW offers some lessons for other community health services aiming to improve service responses to family adversity. Our study defined “identification” as practitioners directly asking about adversity as well as actively listening to issues raised by caregivers to capture a relational approach to care (21). Relational practice is incorporated into the training and holistic assessment practices of social care (e.g., social workers) and allied health providers who also typically have longer consultation time with families than medical professionals (22). The NSW service largely comprised social care practitioners whereas most caregivers in Victoria accessed a clinic staffed by GPs, who face time constraints in practice and so may avoid discussing complex health and social issues that risk opening “‘*Pandora's box' without a process or partners that can effectively address the issues that come to light*” (23). This may be particularly the case in the Victorian service in which, as a Medicare bulk billing GP practice, GPs may be the first port of call for families with numerous and complex needs.

In addition to workforce composition, integrated service infrastructure in NSW may have also supported identification of adversity. Prior to data collection, practitioners in NSW had been afforded training and capacity building opportunities in family partnerships models of practice through SLHD Community Health Services. For families identified as experiencing adversity, referral pathways to the HHAN service embedded within the district existed prior to this study, to provide care coordination and to promote clinical and organizational integration in the broader intersectoral context in which the Marrickville community health service sits (18, 24). As a result, several NSW staff had good knowledge of the referral pathways and standard operating procedures of care coordinators from adjacent health services. This may have removed one of the known barriers to NSW practitioners asking about adversity, i.e., lack of knowledge about available supports. These service pathways were not mapped in Victoria at the time of data collection.

System constraints are a likely contributor to the lack of identification of, and response to, adversity in the Victorian community health service. The parameters and associated reporting requirements of Victorian government child and family community health funding is not easily oriented toward the preventative and holistic approach required to better respond to adversity (25). Recent Victorian government initiatives emerging from the recommendations of the Royal Commission into Victoria's Mental Health System such as Early Help and the Infant, Child and Family Health and Wellbeing Hubs are attempts to reorient community health service provision toward prevention and promotion. However, the funding arrangements to support implementation are yet to be determined.

A final explanation for the improved detection of, and response to, adversity in the NSW service may be because the families accessing the NSW service had significantly higher education levels, were more likely to be born in Australia and less likely to be the lowest SES relative to caregivers accessing the Victorian service. As such, as a cohort, it is plausible that they had higher levels of health and health system literacy, which improved their access. This reflects the “inverse care law” such that that the families more in need were less likely to access care (26). Our finding that many families experiencing adversity are not asked about these experiences suggests that the families identified as experiencing adversity may be the “tip of the iceberg.” For example, although certain adversities are over-represented in families living in low socioeconomic conditions, adversities such as parental mental illness and maladaptive parenting can affect all families (27, 28). Legal needs—captured as societal adversities in our study—were less likely to be identified and responded to in both sites. This reflects the misunderstanding of legal needs seen in the general Australia population (there is no extant research on knowledge of legal issues amongst health practitioners) (29). “Practical problems” (e.g., fines, housing notices, etc) often go unaddressed and so can dominate clinical encounters, lead to missed appointments, disrupt the practitioner-client relationship and worsen health outcomes (30). Health-justice partnerships have the potential to link health consumers with legal assistance for these practical problems to mitigate the impact of societal adversities on family mental health and wellbeing (31).

Given the under-detection of societal adversity in our study, the findings support recommendations for universal approaches to the identification of adversity, with clear pathways to respond to these adversities. However, care should be taken to avoid screening for adversity as a tick box clinical exercise without referral pathways and service partnerships to enable practitioners to appropriately respond to family adversity. Two recent systematic reviews have found no robust evidence that screening for adversity leads to better service access or improved child health outcomes (10, 11). Several studies have also pointed to the potential for screening for adversity to be emotionally difficult and/or retraumatising for families (10). Some authors propose assessing resilience factors alongside adversities to capture a holistic and strengths-based snapshot of family wellbeing (32). More research is required to demonstrate the best way to identify and respond to a range of adversities in community health settings.

### 3.1. Study limitations

Our study has several limitations. Participants reported their experiences within domains of adversity rather than individual types of adversity. This methodological decision was made through consultation with families and a peer researcher to reduce any potential stigmatizing impacts on caregivers, and to avoid replicating a clinical screening process and then failing to provide caregivers with necessary support. While the categorization of these domains was based on theory (1), our categorization of adversities may not represent how caregivers themselves experience or conceptualize adversity. This study also did not measure family strengths which might mitigate the impact of any adversities experienced (32).

Caregivers retrospectively reported their service experiences which introduced the risk of recall bias. This may mean that the primary outcomes may not reflect actual practitioner behavior. However, consistent with the idea of people-centered health systems, caregivers' understandings and experiences of identification of, and responses (or lack thereof) to, adversity are crucial in building and strengthening integrated service delivery (33). Finally, only caregivers with English language fluency could participate which may have excluded caregivers from culturally and linguistically diverse backgrounds. However, 62% of Victorian caregivers were born outside of Australia and 41% spoke a language other than English at home which went some way to rectifying this limitation.

### 3.2. Implications for practice and research

Our findings highlight the need to invest in models of care that improve the identification of, and response to, family adversity in community health services. Integrated health and social care Hubs have the potential to overcome some of the known barriers to identifying and responding to adversity. Hubs models of care are a key recommendation to improve mental health in the 2021 National Children's Mental Health and Wellbeing Strategy (34) and the 2021 Royal Commission into Victoria's Mental Health System (35). Hubs aim to provide a “one stop shop” and holistic multi-disciplinary care for families with complex health and social needs, with potential for more efficient arrangement and use of service resources than presently exists (36).

As part of the broader project, the two community health services in this study are now implementing Hubs including integrative professional support and collaborative practice opportunities through co-location and clinical integration of interdisciplinary staff (13, 25). The Hubs are also providing clearer service linkages and referral pathways for both families and practitioners. Both Hubs are also implementing a wellbeing coordinator role that involves care navigation and social prescribing (i.e., linkages to social and/or community services) based on international evidence that these roles may increase service linkages for families and practitioners and help reduce social isolation, a key contributor to poor mental health outcomes (37, 38). In addition, a health-justice partnership between the community health service and local legal services is being piloted at both Hubs to address the gap around identification or, and response to legal needs detected in this study. Based on existing research, it is theorized that these Hubs may offer a non-stigmatizing “front door” to a range of holistic services for families experiencing adversity e.g., health, financial, child and family, legal services (39).

The detected differences in identification of, and response to, adversity between the two services in our study supports realist-informed research to co-design, test, evaluate and scale Hub models in existing community health services (13, 40). A realist approach incorporates the historical and current service contexts into understandings of for whom, how and why adversities are detected and responded to Pawson and Tilley (41). Despite both study sites being selected for serving families experiencing disadvantage, the study found different patterns of identification of, and response to, adversity that may be explained by the sociodemographic compositions of each community, service infrastructure and workforce composition. The influence of service context detected in this study also highlights the need to incorporate contextual factors into the implementation of broad programs in health settings (e.g., primary care, antenatal care) around detection and response to family violence (42) and financial need (43).

## 4. Conclusion

If Australia is to optimize the mental health and wellbeing of children and families, we need to better identify and respond to family adversities. Our study suggests that families using community health services are experiencing high rates of adversity that are not systematically identified or addressed. With large amounts of funding going to the provision of direct mental health services (35), we are missing an opportunity to support families early in a child's life and earlier in their healthcare journey by better detecting and responding to family adversities. Policy and practice directives support a “one stop shop” Child and Family Hub (34, 44) to provide earlier mental health care. Hub models may be ineffective if they do not also address family adversities as an upstream determinant of mental health. Addressing family adversity will require significant workforce training, organizational culture that supports detection and response to adversities, clear referral pathways and integrated health and social sector responses to a wide range of adversities experienced by Australian families.

## Data availability statement

The raw data supporting the conclusions of this article will be made available by the authors, without undue reservation.

## Ethics statement

The studies involving humans were approved by Ethical approval was received from the Royal Children's Hospital Human Research Ethics Committee (HREC/62866/RCHM-2020) and Sydney Local Health District (HREC/622866/RCHM-2020, 2020/STE05572) for the Victoria and NSW sites, respectively. The studies were conducted in accordance with the local legislation and institutional requirements. Written informed consent for participation in this study was provided by the participants' legal guardians/next of kin.

## Author contributions

TH was involved in study design and concept development, data analysis, interpretation of the findings, and drafted the manuscript. LC was involved in study design and concept development, data collection for Victoria, data analysis, interpretation of the findings and manuscript preparation. SL, SH, SG, RJ, AR, MY, SW, AM, KD, CB, GP, JF, SF, VA, RH, LS, JE, and HH were involved in study design and concept development, interpretation of the findings and manuscript preparation. NS and HL were involved in study design and concept development, data collection for Victoria, interpretation of the findings and manuscript preparation. All authors read and approved the final manuscript.
